# Response Coordination Emerges in Cooperative but Not Competitive Joint Task

**DOI:** 10.3389/fpsyg.2018.01919

**Published:** 2018-10-09

**Authors:** Francesca Ciardo, Agnieszka Wykowska

**Affiliations:** ^1^Istituto Italiano di Tecnologia, Genoa, Italy; ^2^Luleå University of Technology, Luleå, Sweden

**Keywords:** response coordination, shared representations, joint Simon effect, cooperation, competition

## Abstract

Effective social interactions rely on humans’ ability to attune to others within social contexts. Recently, it has been proposed that the emergence of shared representations, as indexed by the Joint Simon effect (JSE), might result from interpersonal coordination (Malone et al., 2014). The present study aimed at examining interpersonal coordination in cooperative and competitive joint tasks. To this end, in two experiments we investigated response coordination, as reflected in instantaneous cross-correlation, when co-agents cooperate (Experiment 1) or compete against each other (Experiment 2). In both experiments, participants performed a go/no-go Simon task alone and together with another agent in two consecutive sessions. In line with previous studies, we found that social presence differently affected the JSE under cooperative and competitive instructions. Similarly, cooperation and competition were reflected in co-agents response coordination. For the cooperative session (Experiment 1), results showed higher percentage of interpersonal coordination for the joint condition, relative to when participants performed the task alone. No difference in the coordination of responses occurred between the individual and the joint conditions when co-agents were in competition (Experiment 2). Finally, results showed that interpersonal coordination between co-agents implies the emergence of the JSE. Taken together, our results suggest that shared representations seem to be a necessary, but not sufficient, condition for interpersonal coordination.

## Introduction

As social species, humans are skillful in attuning to others in social contexts. Several studies showed that performing a task individually could be affected by social presence. Indeed, when embedded in the social environment, we dynamically coordinate our actions with those of others in time and space (Sebanz et al., 2006; Knoblich and Sebanz, 2008). Such coordination during joint actions is supported by a complex plethora of mechanisms, such as shared representations, sensorimotor coordination, and goal sharing (see Vesper et al., 2017 for a review). However, since these mechanisms have been mostly investigated independently, it is still unclear how they are orchestrated in order to support efficient joint tasks.

### Shared Representations and the Joint Simon Effect

According to the shared representations account, joint action is based on the ability to share task representations, i.e., the ability to represent the task as shared, and to create a representation of the task that includes both our and co-agents’ actions (Sebanz et al., 2006). In the recent years, researchers investigated joint action by means of the “Joint Simon” task (Sebanz et al., 2003). In the standard Simon task, participants respond to a non-spatial feature (e.g., color or shape) of stimuli presented to the left or to the right of fixation with assigned right and left key presses. The Simon effect (SE) refers to the finding that performance is faster and more accurate when stimulus and response location spatially correspond, as compared to when they do not (Simon and Rudell, 1967; see also Proctor and Vu, 2006 for a review). The SE is absent when participants perform a go/no-go version of the task, responding only to one feature while withholding the response for the other feature, which indicates that the SE is due to the activation of automatic links between stimulus location and the corresponding response position (Tagliabue et al., 2000). Sebanz et al. (2003) showed that SE occurs even when the Simon task is shared between two participants, i.e., when two participants perform the go/no-go task in a joint context, each one responding to one color only. The spatial compatibility effect emerging in the joint go/no-go task is known as the Joint Simon Effect (JSE)^1^. According to Sebanz et al. (2003, 2006), the JSE has been interpreted as an indication that when people perform together complementary parts of a task, they tend to represent the whole task and to integrate both their and other’s action options into a shared representation, as if they were performing the standard Simon task alone, i.e., performing the task with two hands. In the absence of such a representation, no alternative action is represented and thus no conflict between alternative responses would arise, as is the case of the individually performed go/no-go Simon task. Thus, the JSE has been considered as an index of emergence of shared representations (e.g., Sebanz et al., 2006, 2003; Knoblich and Sebanz, 2008). However, studies that systematically investigated how social presence influences individual performance suggested that when we perform a task along with another person the representations guiding joint performance might differ from representations guiding performance in the individual task (e.g., Ferraro et al., 2011; Ciardo et al., 2016). Several alternative accounts have been proposed to explain the emergence of the JSE (see Prinz, 2015 for a review), including the referential coding account (Dolk et al., 2011, 2013; Dittrich et al., 2013; see Dolk et al., 2014 for a review). The referential coding account proposes that during a joint Go/Nogo Simon task, the presence of any salient action event, generated by a biological or non-biological agent (Stenzel and Liepelt, 2016; Miss and Burkhart, 2018), is represented by an action event code. Given the high similarity of the two action events in the Joint Simon task (i.e., pressing a button), participants need to discriminate between internally (one’s own) and externally (the other agent’s) activated events. In order to solve the conflict arising from this discrimination, the differences between the two action events (i.e., the left-right location of the response), are strengthened and automatically interfere with the task-irrelevant stimulus spatial code, which generates the JSE (Dolk et al., 2014).

### Interpersonal Coordination

Sharing the context with another agent does not always require *intentional* representation of one’s own and others’ actions. Indeed, the presence of another person can interfere with our performance at a lower level, as in the case of sensorimotor timing (e.g., Schmidt and Turvey, 1994; Richardson et al., 2007). For example, during a conversation, we tend to nod at a same rhythm as the speaker. Similarly, when we walk with someone, we reciprocally adapt our gait to each other. This tendency to unintentionally adapt the timing of our movements to others is called *entrainment* and it seems necessary in order to be temporally coupled with others (Marsh et al., 2009). Entrainment also underlies joint action, according to the dynamic account (see Marsh et al., 2009 for a review). For instance, it has been shown that pairs of participants performing rhythmic movements (i.e., swinging a pendulum or rocking chairs) tend to become temporally correlated by adopting the same movement rate (Schmidt and Turvey, 1994; Richardson et al., 2007). Vesper et al. (2011) showed that when pairs of participants perform two independent Simon tasks at the same moment, their responses tend to be coordinated (Vesper et al., 2011). Specifically, response variability positively correlated with asynchrony in reaction times across the two members of the pairs, suggesting that reducing response variability may represent an implicit strategy to facilitate cooperation. Similarly, other results show that coordination supports access to others’ mental states and spontaneous cooperation (Semin and Cacioppo, 2008; Koehne et al., 2016). In a recent study, Malone et al. (2014) investigated the dynamic structure of reaction times (RTs) in a Joint Simon task. The authors compared the response variability structure of participants performing a go/no-go Simon task. Two groups of participants performed the same go/no-go Simon task individually or together with another person having the complementary go/no-go assignment. Results showed that variability structure was whiter^2^ in the individual than in the joint condition (Malone et al., 2014); indicating that when participants performed the task side-by-side of another person, responses were characterized by nested patterns of variability which were not due to random fluctuations (Malone et al., 2014). In line with the idea of decreasing fractal structure of RT variability, the authors reported that RTs of pairs in the joint condition were more correlated across time scales than RTs of pseudo-pairs of participants who performed the task individually. These latter results suggested that responses of co-agents during the joint Simon task were coupled and that the dynamics of co-agents’ responses might be mutually constrained. In sum, the authors proposed that “*dynamic processes of constraints may decouple behavior over time”* (cf. p. 6 Malone et al., 2014) and may underlie the JSE instead of any form of shared or integrated representation of the task. Alternatively, it is also plausible that the emergence of shared representations, or the integration of “self” and “other” action events, may actually promote emergent temporally evolving coupling and modulate the inter-agent response dynamics.

### Social Context and Shared Representations: The Case of Cooperation and Competition

Cooperation and competition are social relations that rely on opposite goal interdependency (Deutsch, 2011), and differently affect social cognitive processes, as joint attention (e.g., Ciardo et al., 2015), sensorimotor synchronization (e.g., Fairhurst et al., 2012), and reach-to-grasp kinematics (e.g., Ciardo et al., 2017). When we cooperate with someone, our goals are positively related. In contrast, when we compete, reaching our personal goal is negatively related to others’ achievement of the goal: if our competitor reaches his/her goal, then we cannot reach our goal anymore (Deutsch, 2011). Positive and negative goals interdependency between co-agents differently affects the emergence of shared representations (e.g., Ruys and Aarts, 2010; Iani et al., 2011, 2014) and self-other integration (Hommel et al., 2009; Ruissen and de Bruijn, 2016). For instance, Hommel et al. (2009), manipulated the valence of the interaction between two co-agents during a Joint Simon Task. Participants performed the task with a friendly and cooperative, or with an intimidating and competitive confederate. Results showed that the JSE occurred only for participants involved in a positive relationship, whereas the negative relationship led to a reduction of the JSE. Similarly, Iani et al. (2011, 2014) showed that when pairs of participants performed a joint Simon task, the JSE emerged only when the two co-agents were required to cooperate but not when they were in competition against each other (Iani et al., 2011, 2014). Under the cooperative condition, participants were told that the pair with the fastest and most accurate responses would receive a reward. This condition elicited a positive interdependence, as the success of one individual rendered the success of the other more likely. Under the competitive condition, they were told that the participant of the pair with the fastest and most accurate responses would receive a monetary reward. Such a design indicated that by manipulating goals interdependency, it is possible to promote or inhibit the emergence of shared representations without manipulating the physical and dynamical features of the social environment and its task constraints. According to the referential coding account (Dolk et al., 2013, 2014), the lack of JSE during competitive tasks can be explained by the fact that negative interpersonal relationships do not promote self-other integration. Thus, during competitive tasks participants do not need to discriminate between internally and externally activated action events, and they do not need to strength task-relevant information (i.e., left and right response location) resulting in the lack of the JSE. Results from a recent study by Ruissen and de Bruijn (2016) are in line with the self-other integration account (Dolk et al., 2014) showing that the JSE is reduced following a competitive game play. According to Ruissen and de Bruijn (2016), motivation and contextual factors might affect self-other integration during the Joint Simon task by exerting different effect on attentional processes. In a cooperative situation we might be motivated to attend to our co-agents performance even if, as in the Joint Simon task (Ferraro et al., 2011), it actually interferes with our own performace, – in order to monitor potential co-agent’s mistakes, and to better adapt our internal action model. On the contrary, during competitive interactions, co-agents might be focused on stabilizing their own performance and do not attend the co-agent’s behavior, which results in attenuation of self-other integration (Hommel et al., 2009; Ruissen and de Bruijn, 2016).

Recently, Keller et al. (2016) proposed a model of joint action, which connects shared representation of goals and interpersonal coordination. The authors proposed that during joint action, distinct self and other internal models are maintained in order to ensure that each co-agent controls their action planning and execution. When shared representations of goals are established, self and other models work together allowing co-agents to anticipate, attend, and adapt to each other in real time (Keller et al., 2014). The coupling of self and other models into a joint model facilitates interpersonal coordination. Thus, the emergence of shared representations of goals guide joint action by supporting the interaction between cognitive and online sensorimotor processes. Previous studies investigating how cooperation and competition affect self-other integration or shared representations used a monetary reward to manipulate cooperation and competition between co-agents (Ruys and Aarts, 2010; Iani et al., 2011). However, individual and contextual differences can shape the actual perception and experience of a monetary reward as a motivational cue (e.g., Kahneman and Tversky, 1979; see Schultz, 2006 for a review). This would explain the controversial nature of the results reported by previous studies on how competition affects JSE (Ruys and Aarts, 2010; Iani et al., 2011). In order to minimize the effect of individual and contextual differences in the motivation to cooperate or compete, in the present study, we manipulated positive and negative interdependency between co-agents through punishment avoidance. Indeed it has been shown that reward and punishment avoidance emerge from different learning mechanisms rely on distinct neural circuits (e.g., Palminteri et al., 2012). Thus, by using punishment avoidance instead of reward, we aimed at testing whether previous findings showing that the JSE can be modulated by cooperative vs. competitive instructions generalize to different types of experimental manipulation.

### Aim of Study

The present study aimed at examining the relationship between interpersonal coordination and the JSE, with JSE being taken as an index of shared representations. To this end, in two experiments we asked participants to perform a go-no/go Simon task alone or side-by-side of another person. In Experiment 1, we investigated the response coordination when co-agents were required to cooperate, with the assumption that cooperation promotes self-other integration or the emergence of shared representations. In Experiment 2, we examined response coordination when co-agents’ goals were mutually exclusive, like in competition, assuming that in this case, self-other integration would be attenuated, or shared representation would not be activated.

## Experiment 1

The present experiment aimed at assessing interpersonal response coordination during joint action. To this end, we compared the coordination between RTs when participants performed a go/no-go Simon task alone or together with another person. We focused on cooperative joint actions, i.e., when the goals of two co-agents are positively related to each other. In line with previous studies, we expected a non-significant SE (i.e., no difference between corresponding and non-corresponding trials) when participants perform the task alone, and a JSE when they are required to cooperate (Hommel et al., 2009; Ruys and Aarts, 2010; Iani et al., 2011, 2014; Ruissen and de Bruijn, 2016). Regarding response coordination, to explore interpersonal coordination in the context of JSE, we examined if RTs of co-agents were correlated (i.e., coordinated) with each other over time. We hypothesized that if shared representations or self-other integration are reflected in the dynamics of the behavior then the response coordination should be greater between the RT time-series of individuals in the joint condition, as compared to RT time-series of pseudo–pairs created using RT time-series from the two individual conditions. Specifically, a higher percentage of response coordination in RT times-series is expected for the Joint compare to the Individual condition (Malone et al., 2014).

### Materials and Methods

#### Participants

Twenty participants (11 males; 4 left-handed; Mean age: 24 ± 3.9 years) took part in the study. All participants had normal or corrected-to-normal vision and were not informed with respect to the purpose of the experiment. Participants received a reimbursement of 15€ for their participation. All gave their written informed consent before participating. Both Experiment 1 and Experiment 2 were conducted in accordance with the ethical standards laid down in the 2013 Declaration of Helsinki and were approved by the local ethical committee (Comitato Etico Regione Liguria). Sample size was defined according to previous experiments (Ruys and Aarts, 2010; Iani et al., 2011), and by an a priori power analysis indicating a sample *N* = 18 to detect a medium effect size [Cohen’s d for repeated measures (Dz) = 0.60, alpha (one-tailed) = 0.05 and power = 0.95] for within-subjects comparisons. Participants were recruited individually from the subject database of the Italian Institute of Technology. They were paired according to the time slots in which they were available to take part in the experiment.

#### Apparatus and Stimuli

Stimuli presentation, response timing, and data collection were controlled by the E-Prime version 3 software (Psychology Software Tools, Inc.). Stimuli were red and green solid squares (2.3°× 2.3°), which were randomly presented on the left or on the right of a central white fixation cross (0.6°× 0.6°) on a black background. Responses were executed by pressing with the index finger the “z” or “-” key of a standard Italian QWERTY keyboard. Response keys were highlighted with two white circular stickers. The experiment was carried out in a dimly lit and noiseless room. Participants were seated facing a 27″ LCD screen driven by a 2.4 GHz processor computer. Viewing distance was about 60 cm.

#### Procedure

Pairs of participants performed two consecutive sessions, separated by a 5-min interval and lasting about 60 min in total. To avoid transfer of learning effects typical of spatial compatibility tasks (Ansorge and Wühr, 2009; Dittrich et al., 2012; Lugli et al., 2013), the order of the two sessions was fixed: an Individual session was followed by a Joint session (for a similar procedure see Dittrich et al., 2017). In the Individual session, each participant performed the task alone, sitting to the right or on the left from of the center of the screen, with an empty chair next to him/her. Left-handed participants seated always on the left side of the screen, in order to let them perform the task with their dominant hand. At their arrival to the lab, participants were told that they were going to perform two different experiments. The two members of the pair participated in the Individual session in parallel (i.e., at the same time), sitting in two different rooms without any possibility to see or talk to each other. Instructions for the individual session were provided separately to each participant by the same experimenter. In the Joint session, participants seated side-by-side, one to the left and one to the right of the center of the screen. Pairs of participants were instructed to cooperate in order to be the best-performing pair, in terms of both speed and accuracy. They were told that, at the end of the experiment, if they did not perform as the best couple they would receive a punishment, consisting in performing an additional task (i.e., performing the first session again). In both sessions (i.e., Individual and Joint), the experimental procedure was as follows: A trial began with the presentation of the fixation cross at the center of the screen. After 1 s, the stimulus appeared to the right or to the left of the fixation and remained visible until a response was collected, or for 800 ms. Maximum time allowed for response was 1 s after stimulus presentation. Immediately after a response was collected, or the stimulus elapsed, a black screen was presented for 1 s. In the Individual session, Nogo stimulus was presented for 800 ms and followed by a 1 s black screen before the next trial started. For both sessions, the task consisted of 16 practice trials and 384 experimental trials divided into four blocks of 96 trials each. For half of the trials, stimulus and response location corresponded (corresponding trials), for the other half, they did not correspond (non-corresponding trials). A fictional partial score was displayed at the end of each block. The score was computed as the difference between corresponding and not-corresponding trials. Participants were told that the score was computed by an algorithm based on their speed in responding, corrected by the overall percentage of correct answers. For half of the pairs, the participant sitting on the right chair pressed the right key to the red stimulus whereas the participant sitting on the left chair pressed the left key to the green stimulus. The other half was assigned opposite stimulus–response mapping. Response-, seat- and stimulus assignment to each participant was identical across the two sessions.

### Data Analysis

First, we analyzed correct responses to check the JSE. Mean correct RTs were submitted to a repeated-measures analysis of variance (ANOVA) with Condition (Individual vs. Joint), and Correspondence (non-corresponding vs. corresponding) as within-subjects factors.

In order to evaluate whether JSE requires time to emerge, we conducted a distributional analysis of RTs (Ratcliff, 1979). To have enough observations in each bin, we chose to divide the RT distribution in quartiles (Liepelt et al., 2011). Thus, individual correct RTs for each condition were rank ordered and divided into four bins. Mean RTs for each bin were then entered into a repeated-measures ANOVA with Condition (Individual vs. Joint), and Correspondence (non-corresponding vs. corresponding) and Bin (1–4) as within-participant factors. To investigate trial-by-trial modulations (Liepelt et al., 2011; Yamaguchi et al., 2018), mean RTs were submitted to an ANOVA with Condition (Individual vs. Joint), Trial Transition (n-1 Go/ n go vs. n-1 Nogo/ n go), Trial n-1 Correspondence (non-corresponding vs. corresponding), and Trial n Correspondence (non-corresponding vs. corresponding) as within-participant factors. When necessary, comparisons were performed using paired samples *t*-tests. Significance thresholds were corrected for the number of comparisons (Bonferroni correction).

To quantify the degree of coordination between the agents, following study Malone et al.’s (2014), we applied instantaneous cross-correlation on RTs series (Barbosa et al., 2008); which allows determining the correlation between time-series across multiple time-scales. This is done by computing correspondence between two time-series recursively and generating a time-series of how past and future samples are correlated at all points in time. This method has been applied to determine objective coordination between non-synchronous behaviors occurring at different time lags, like in articulatory coordination of two vocals tracts (Vatikiotis-Bateson et al., 2014). Subsequently, an index of response coordination was estimated as the proportion of correlated activity (i.e., the proportion of *r* > 0.25, see Malone et al., 2014, 2013) between the RT time-series of the two members of a pair. RT time-series were computed by ordering for each participant RTs in the order they were collected, and then by subtracting from each data point the mean of respective condition for each participant. RTs for missing and incorrect responses were substituted by the mean of RTs for the respective condition. We ran the instantaneous correlation analysis for offsets of -9 to + 9 trials with a conservative (η = 0.1) non-causal filter (Barbosa et al., 2008). Thus, the offset range was chosen by reducing the interval size applied in Malone et al.’s (2014) study, in order to consider delays proportional to the lower number of trials.

Finally, a paired samples *t*-test was applied to compare if the proportion of correlated response activity (i.e., index of response coordination) within each pseudo-pair in the individual condition differed from the percent of coupling observed for pairs in the joint condition.

### Results

#### Reaction Times

**FIGURE 1:**
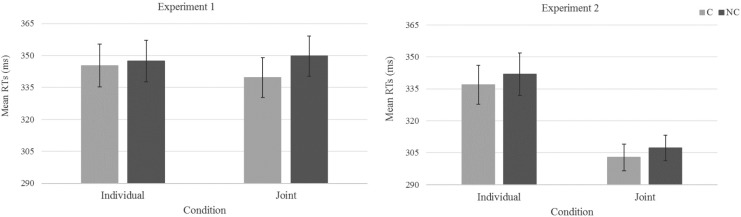
Mean reaction times (ms) as a function of Condition (individual vs. joint) and Correspondence (non-corresponding vs. corresponding) in Experiment 1 (Left panel) and Experiment 2 (Right panel). Error bars show standard errors of the means.

Errors were 0.4 and 0.5% of the total amount of trials, for the Individual and Joint conditions, respectively, and were not further analyzed. Tukey outlier thresholds (1977) were used for each condition to identify outliers in the number of erroneous trials. No participants were excluded. Mean RTs are summarized in **Table 1**. The ANOVA revealed a main effect of Correspondence, *F*_1,19_ = 8.70, *p* = 0.008, $\eta_{p}^{2}$ = 0.31, together with a significant two-way interaction with Condition, *F*_1,19_ = 15.09, *p* = 0.001, $\eta_{p}^{2}$ = 0.44. Pairwise comparisons showed that the difference between corresponding (*M* = 340 ms) and non-corresponding trials (*M* = 350 ms) was significant for the Joint condition only, *t*_19_ = 4.11, p_Bonferroni–corrected_ = 0.001, *d* = 0.92. In the Individual session no effect of correspondence was evident (*M* = 345 and *M* = 347 ms for corresponding and non-corresponding trials, respectively), *t*_19_ < 1 (**Figure 1**).

**Table 1:** Experiment 1: Mean correct reaction times (and standard deviation) in ms as a function of Condition (individual vs. joint) and Correspondence (non-corresponding vs. corresponding).

	Individual	Joint
NC	347_ (44)_	350_(42)_
C	345_(45)_	340_(42)_


#### RTs Distribution

**FIGURE 2:**
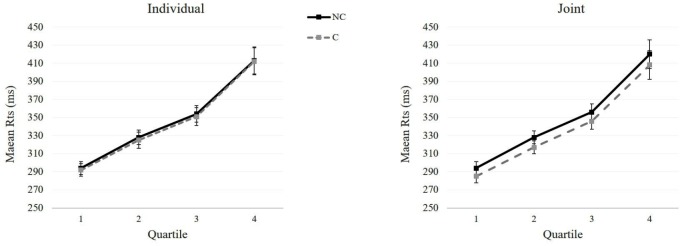
Experiment 1: Mean reaction times (ms) as a function of Correspondence (non-corresponding vs. corresponding) across quartiles in the Individual (Left panel) and Joint condition (Right panel). Error bars show standard errors of the means.

Besides the main effect of Correspondence and its interaction with Condition already reported in the previous analysis, the ANOVA revealed a main effect of Bin, *F*_1,19_ = 175.98, *p* < 0.001, $\eta_{p}^{2}$ = 0.90, indicating that RTs increase across quartiles. No other main effects or interactions were significant, all *p*s > 0.77 (**Figure 2**).

#### Trial-by-Trial Modulation and Transition Effects

The first trial of each block, errors and responses that were preceded by an incorrect response were discarded from the analysis (1.15 and 1.41% of the total trials in the Individual and Joint condition, respectively). The results are summarized in **Table 2**. The ANOVA showed that responses were faster in corresponding (*M* = 342 ms, *SE* = 8.74 ms) than non-corresponding (*M* = 348 ms, *SE* = 8.71 ms) trials, as indicated by the main effect of Trial n Correspondence*, F*_1,19_ = 9.66, *p* = 0.006, $\eta_{p}^{2}$ = 0.34. As in the previous analysis, the two-way interaction between Condition and Correspondence was significant, *F*_1,19_ = 8.73, *p* = 0.008, $\eta_{p}^{2}$ = 0.32. Pairwise comparisons showed a significant 9-ms JSE for the Joint condition only, *t*_19_ = 3.98, p_Bonferroni–corrected_ < 0.001, *d* = 0.89, and a non-significant 3-ms JSE in the Individual session, *t*_19_ = 1.37, p_Bonferroni–corrected_ = 0.187, *d* = 0.31. The interaction between Trial n Correspondence and Trial n-1 Correspondence was also significant, *F*_1,19_ = 58.88, *p* < 0.001, $\eta_{p}^{2}$ = 0.32. *Post hoc* comparisons showed a 15-ms effect after a corresponding n-1 trial, *t*_19_ = 6.4, p_Bonferroni-corrected_ < 0.001, *d* = 1.43, and a non-significant 3-ms effect after non-corresponding n-1 trials, *t*_19_ = 1.11, p_Bonferroni-corrected_ > 0.05, *d* = 0.25. The three-way interaction between Trial Transition, Trial n-1 Correspondence, and Trial n Correspondence was significant, *F*_1,19_ = 18.49, *p* < 0.001, $\eta_{p}^{2}$ = 0.49. Planned comparison showed that trial-by-trial modulation occurred always for Nogo/go transitions with a significant 24-ms effect following a corresponding n-1 trial, *t*_19_ = 5.63, p_Bonferroni-corrected_ < 0.001, *d* = 1.26, and a reversed 9 ms effect following a non-corresponding n-1 trial, *t*_19_ = 3.01, p_Bonferroni-corrected_ = 0.007, *d* = 0.67. On the contrary, no trial-by-trial modulations occurred for Go/go transitions, all *p*s > 0.06. No other main effects or interaction were significant, all *p*s > 0.14.

**Table 2:** Experiment 1: Mean correct reaction times (and standard deviation) in ms as a function of Trial Transition (Nogo/go, Go/go), Trial n-1 (corresponding, C vs. non-corresponding, NC), and Trial n (corresponding, C vs. non-corresponding, NC).

Nogo/go transitions				Go/go transitions			
	Trial n				Trial n		
Trial n-1	C	NC	SE	Trial n-1	C	NC	SE
C	334_(41)_	358_(42)_	24	C	342_(43)_	348_(38)_	6
NC	350_(40)_	341_(41)_	-9	NC	341_(37)_	345_(37)_	4


*The Simon effect (SE) is computed as the difference in RTs between non-corresponding and corresponding trials.*

#### Response Coordination

As mentioned above, we compared the proportion of response coordination (i.e., the proportion of correlation between the times series higher than 0.25) within each pseudo-pair (*N* = 10) in the individual condition with the percent of response coordination observed for pairs in the joint condition. Results showed higher response coordination for the Joint (15.3%) than for the Individual session (12.7%), *t*_9_ = 3.44, *p* = 0.007, *d* = 1.09.

### Discussion

In Experiment 1, we examined coordination of RTs when participants performed a go/no-go Simon task alone or side-by-side of another person, in a cooperative context. In line with previous studies, results from mean RTs showed a non-significant SE when participants performed the task alone, and a significant JSE when they were performing the task side-by-side and were instructed to cooperate (Iani et al., 2011, 2014, see Karlinsky et al., 2017b for a meta-analysis on the magnitude of the JSE). The comparison of the distributional trends showed that response speed did not affect the magnitude of the SE neither in the Individual task nor in the Joint task. The similarity in the distributional patterns between the Joint and the Individual tasks replicates previous results reported by study Liepelt et al.’s (2011), suggesting that the emergence of JSE cannot be attributed to different temporal dynamics underlying the two conditions. Trial-by-trial modulations occurred both in the Individual and Joint conditions, as indicated by the lack of significant interaction involving trial sequence (n-1/n) and Condition. As reported by previous studies (Liepelt et al., 2011; Yamaguchi et al., 2018), trial-by-trial modulations occurred when Go trials were proceeded by a Nogo trial (Nogo-Go Transition), probably reflecting response inhibition during Nogo trials. Trial-by-trial modulations mimic the pattern typically reported in standard two-choice Simon tasks (Iani et al., 2009; Ciardo et al., 2018), with a reversed effect following non-corresponding n-1 trial and a positive effect following corresponding n-1 trial. These trial-by-trial modulations have been taken as evidence that the conflict experienced in a trial is accompanied by changes aiming at preventing the reocurrence of the conflict in the next trial by means of enhanced processing of task-relevant information (e.g., Egner and Hirsch, 2005) or inhibition of task-irrelevant features (e.g., Ridderinkhof, 2002). Alternatively, it has been proposed that trial-by-trial modulation may reflect binding effects (e.g., Hommel et al., 2004). Indeed in the Simon task, sequences of two corresponding trials (C–C) and sequences of two non-corresponding trials (NC–NC) are either complete repetitions or complete changes of stimulus position and response or complete changes of both stimulus position and response. In contrast, mixed sequences (C–NC or NC–C) are always partial repetitions in which either stimulus position or response repeats. Thus, the SE may be reduced following a non-corresponding trial because responses are faster for complete repetitions and alternations compared to partial repetitions (Hommel et al., 2004). The results of response coordination showed that when co-agents were instructed to cooperate, their RT time-series were more coordinated (i.e., a higher percentage of correlation) with each other over time, as compared to when they were performing the task individually. Such result confirms and extends Malone et al. (2014) evidence for the idea that coordination of behavior is observed together with the JSE.

## Experiment 2

Experiment 1 suggested that when co-agents’ goals are positively related, the correlation between co-agents’ responses across time scales increases. However, it could be that co-agents’ coordination reflects their adaptation in space and time related to any dynamic event occurring during the task, like another agent (human or not) acting in the same environment, independently from positive goal. Thus, it is possible that the increase in response coordination reported in Experiment 1 results from the natural tendency to adapt the timing of our movements to external events (e.g., Marsh et al., 2009), rather than resulting from integration of self and other action events, or from the emergence of a shared representation. In line with this hypothesis, there are several results showing that the JSE can occur even when no shared representation is necessary, like when the co-agent is not present physically (e.g., Sellaro et al., 2013) or when an object is performing the complementary go/no-go task (Stenzel and Liepelt, 2016). For example, a recent evidence showed that JSE emerges even when the alternative response is executed by a non-human agent (e.g., a Japanese cat, a metronome, or a wooden hand, Dolk et al., 2013; Stenzel and Liepelt, 2016). Interestingly, the JSE was larger when the external event (i.e., the non-human agent) was acting in a turn-taking way with respect to the participant, as compared to a condition when it was acting in a continuous way, i.e., not task-related (Stenzel and Liepelt, 2016). To test this alternative explanation, in Experiment 2 we examined the effect of mutually exclusive goals (assuming no shared representation) on response coordination. As in Experiment 1, we compared coordination between RT time-series when participants performed a go/no-go Simon task alone or side-by-side of another person. However, during the Joint session participants were instructed to compete against each other. Note that competition is a particular case of joint task in a shared environment, where individuals work to reach an individual goal that – in order to be reached – excludes the goal of the other co-agent. In line with previous studies showing that competition disrupts the emergence of shared representation in joint tasks (Ruys and Aarts, 2010; Iani et al., 2011, 2014) or affect the integration of self and other action events (Hommel et al., 2009; Ruissen and de Bruijn, 2016); we expected no difference in the SE between the Individual and Joint condition. Regarding response coordination, we hypothesized that if findings of Experiment 1 are merely due to environmental perturbations produced by dynamic events, i.e., the co-agent acting in the shared environment, then results should replicate the pattern reported in Experiment 1, with greater response coordination (i.e., higher percentage of correlation) in the joint condition compared to the individual condition. This result would speak against the idea that shared representations, are the consequence of response coordination. On the contrary, if the percentage of response coordination reflects the emergence of shared representations, or the integration of self-other action events, then the social presence should not modulate coordination between RT times-series across the individual and joint conditions, similarly to the standard SE. This would speak in favor of the hypothesis that interpersonal coordination yields shared representation.

### Materials and Methods

#### Participants

Twenty-six new participants (8 males; 4 left-handed; Mean age: 24 ± 2.9 years), selected as in the previous experiment, took part in Experiment 2. All participants gave their written informed consent and the study was conducted in accordance with the ethical protocol applied also in Experiment 1. Three pairs, six participants in total, were excluded from the data analysis, given the number of errors made by at least one member of the pair.

#### Apparatus, Stimuli, and Procedure

The apparatus, stimuli, and procedure were the same as in Experiment 1. With the only exception that in the Joint session, pairs of participants received the instructions to compete against each another. They were told that at the end of the experiment, the worst performer of the pair would receive a punishment, i.e., s-/he had to perform an additional task. Apart from the instructions, all other aspects of the experimental design were as in Experiment 1.

### Results and Discussion

#### Reaction Times

Errors were 0.6 and 1.1% of the total amount of trials for the Individual and Joint conditions, respectively, and were not further analyzed. Tukey outlier thresholds (1977) were used for each condition to identify outliers in the number of erroneous trials. These thresholds removed 1 participant from the Individual condition and 2 participants from the Joint condition. In total 3 pairs were excluded from the analyses, thus data analysis was run on a sample size including 10 pairs (*N* = 20). Mean correct reaction times (RTs) were analyzed as in Experiment 1. The results are summarized in **Table 3**. The analysis revealed a main effect of Condition, *F*_1,19_ = 34.82, *p* < 0.001, $\eta_{p}^{2}$ = 0.65, indicating that participants performed faster in the Joint condition (*M* = 305 ms) than the Individual condition (*M* = 339 ms). Main effect of Correspondence, *F*_1,19_ = 7.50, *p* = 0.013, $\eta_{p}^{2}$ = 0.28, indicated faster responses for corresponding (*M* = 320 ms) than non-corresponding trials (*M* = 325 ms), however, this effect did not differ across the Joint and the Individual conditions, as indicated by the lack of significance for the two-way interaction, *F* < 1.

**Table 3:** Experiment 2: Mean correct reaction times (and standard deviation) in ms as a function of Condition (individual vs. joint) and Correspondence (non-corresponding vs. corresponding).

	Individual	Joint
NC	342_ (45)_	307_(27)_
C	337_(41)_	303_(28)_


#### RTs Distribution

Besides the main effect of Correspondence and Condition already discussed in the previous analysis, the ANOVA revealed a main effect of Bin, *F*_1,19_ = 528.90, *p* < 0.001, $\eta_{p}^{2}$ = 0.97, indicating that RTs increase across quartiles. Two-way interaction between Condition and Bins was significant, *F*_1,19_ = 20.90, *p* < 0.001, $\eta_{p}^{2}$ = 0.52. Pairwise comparisons showed that for all the quartiles responses were faster in the Joint compared to the Individual condition, all *p*s < 0.001. No other main effects or interactions were significant, all *p*s > 0.55.

#### Trial-by-Trial Modulation and Transition Effects

**FIGURE 3:**
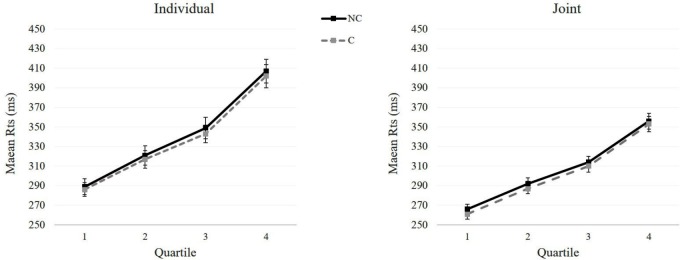
Experiment 2: Mean reaction times (ms) as a function of Correspondence (non-corresponding vs. corresponding) across quartiles in the Individual (Left panel) and Joint condition (Right panel). Error bars show standard errors of the means.

The first trial of each block, errors and responses that were preceded by an incorrect response were discarded from the analysis (1.20 and 1.04% of the total trials in the Individual and Joint condition, respectively). The results are summarized in **Table 4**. The ANOVA showed that responses were faster in corresponding (*M* = 319 ms, *SE* = 7.31 ms) than non-corresponding (*M* = 324 ms, *SE* = 7.61 ms) trials, as indicated by the main effect of Trial n Correspondence*, F*_1,19_ = 6.78, *p* = 0.017, $\eta_{p}^{2}$ = 0.26. As in the previous analysis, the main effect of Condition was significant, *F*_1,19_ = 34.24, *p* < 0.001, $\eta_{p}^{2}$ = 0.64, as well its interaction with Trial n-1 Correspondence, *F*_1,19_ = 6.40, *p* < 0.001, $\eta_{p}^{2}$ = 0.64. The interaction between Trial n Correspondence and Trial n-1 Correspondence was also significant, *F*_1,19_ = 91.40, *p* = 0.020, $\eta_{p}^{2}$ = 0.25. *Post hoc* comparisons showed a 14-ms effect after a corresponding n-1 trial, *t*_19_ = 7.04, p_Bonferroni-corrected_ < 0.001, *d* = 1.58, and a reversed 5-ms effect after non-corresponding n-1 trials, *t*_19_ = 2.55, p_Bonferroni-corrected_ = 0.02, *d* = 0.57. The three-way interaction between Trial Transition, Trial n-1 Correspondence, and Trial n Correspondence was significant, *F*_1,19_ = 54.42, *p* < 0.001, $\eta_{p}^{2}$ = 0.74. Planned comparison showed that trial-by-trial modulations for Nogo/go transitions with a significant 22-ms effect following a corresponding n-1 trial, *t*_19_ = 8.64, p_Bonferroni-corrected_ < 0.001, *d* = 1.93, and a reversed 11-ms effect following a non-corresponding n-1 trial, *t*_19_ = 4.94, p_Bonferroni-corrected_ < 0.001, *d* = 1.11. When trial transition was Go/go a significant 6-ms effect occurred following a corresponding n-1 trial, *t*_19_ = 2.65, p_Bonferroni-corrected_ = 0.02, *d* = 0.59, and a 2-ms non-significant effect following a non-corresponding n-1 trial, *t*_19_ < 1. No other main effects or interaction were significant, all *p*s > 0.36 (**Figure 3**).

**Table 4:** Experiment 2: Mean correct reaction times (and standard deviation) in ms as a function of Trial Transition (Nogo/go, Go/go), Trial n-1 (corresponding, C vs. non-corresponding, NC), and Trial n (corresponding, C vs. non-corresponding, NC).

Nogo/go transitions				Go/go transitions			
	Trial n				Trial n		
Trial n-1	C	NC	SE	Trial n-1	C	NC	SE
C	310_(33)_	332_(358)_	22	C	320_(35)_	326_(35)_	6
NC	327_(31)_	316_(33)_	-11	NC	320_(35)_	322_(36)_	2


*The Simon effect (SE) is computed as the difference in RTs between non-corresponding and corresponding trials.*

#### Response Coordination

Reaction time-series were computed and analyzed as in Experiment 1. A paired samples *t*-test was applied to compare if the proportion of correlated activity between the two members of the pair (*N* = 10) in the individual condition differed from the percent of response coordination observed in the joint condition. Results showed no difference in the proportion of correlated activity between the Individual (12.7%) and the Joint session (13.1%), *t*_9_ < 1.

### Discussion

Experiment 2 aimed at testing if the increased response coordination reported during the joint task in Experiment 1 can be interpreted as the consequence of the mere temporal coupling with external events. To this end, we compared response coordination of participants performing a go/no-go Simon task alone or in competition with another person. Results from mean RTs showed that participants were faster in the Joint condition, as compared to the Individual condition. This result is not surprising since participants were instructed to be the best performer in the couple, in order to avoid punishment. A similar increase in speed of responses has been reported in a recent study investigating the role of turn-taking in the emergence of JSE (Karlinsky et al., 2017a). Specifically, the authors reported faster RTs when the structure of the task did not require to alternate own actions with those of the co-agent. Thus, it is possible that in our experiment the competitive framework affected the perception of turn-taking during the task. In line with our prediction, results from mean RTs indicated no difference in the SE (5 ms) between the Individual and the Joint condition. In line with results from Experiment 1, no difference emerged from the analysis of distributional trends across the Individual and the Joint condition. Similarly, trial-by-trial modulations occurred both in the Individual and Joint conditions. Again, trial-by-trial modulations were stronger for the Nogo/go transitions compared to the Go/go transitions.

Response coordination analysis showed that the percentage of coordination between RT time-series was similar for the Individual and the Joint condition. Results of Experiment 2 suggest that the increase in response coordination reported in Experiment 1 cannot be interpreted as merely the consequence of the perturbation produced by a dynamic event in the task environment. Indeed, if this was the case, a similar pattern should have emerged in Experiment 2. On the contrary, the present experiment shows that, despite the presence of the co-agent acting in a shared environment, participants did not coordinate their responses with those of a competitor.

### Comparisons Between Experiments

#### Linear Mixed-Effects Analysis

To examine the contribution of response coordination in the JSE, we used a linear mixed-effects model analysis on mean RTs to re-analyze data from both Experiment 1 and Experiment 2. We compared our Model 1, which, as fixed factors, included Condition (Individual, Joint), Correspondence (corresponding, non-corresponding) and their interaction, with Model 2, which included coordination as a random effect. We began with a maximal random effects structure (Barr et al., 2013; Bates et al., 2015). Then, we redefined the model by including coordination as a random effect to check whether the goodness of fit was significantly increased or reduced after removing variance accounted by the random effect of coordination. In other words, by using the percentage of correlation between co-agents’ RT time-series as the random effect, we controlled if it influenced main effects. The significance of the effects and parameters was evaluated using Chi-square test. Analyses were carried out using the package lme4 (version 1.0-5; Bates et al., 2015) available for the statistical software R (version 3.0.1, freely available at http://www.rproject.org). Results of the two models are displayed in **Table 5**.

**Table 5:** Model comparisons for the random effect of correlated response coordination on mean RTs.

Experiment	Model	*DF*	*X^2^*	*P*
Exp. 1	Model 1	5	2275.2	<0.001
	Model 2	6		
Exp. 2	Model 1	5	4.5796	0.032
	Model 2	6		


#### Experiment 1

Results showed that including the percentage of response coordination as random effect significantly improved model fit. Then, we re-estimated the mean differences in mean RTs for Experiment 1 using Model 2. Results showed that both the main effect of Correspondence and its interaction with Condition were still significant, *χ^2^* = 19.58, *p* < 0.001 and *χ^2^* = 8.22, *p* = 0.001.

#### Experiment 2

Results showed that including the percentage of response coordination as random effect significantly improved model fit. We then re-estimated the mean differences in mean RTs for Experiment 2 using Model 2. Results showed that only the main effect of Condition was still significant, *χ^2^* = 10.50, *p* = 0.001, while the main effect of Correspondence did not reach the significance, *χ^2^*< 1.

Results from the linear mixed models analysis indicate that in both experiments introducing the percentage of response coordination as random effect increased the goodness of fit. In Experiment 1, by removing the variance explained by response coordination, the main effect of Correspondence survived, and so did the two-way interaction with Condition. Such a result suggests that the significant JSE reported in the joint condition is not fully explained by correlation between co-agents’ responses. In contrast, no main effect of Correspondence emerged in Experiment 2 when the percentage of response coordination is introduced as random effect. Thus, it is possible that the main effect of Correspondence in Experiment 2 could be a false positive (i.e., a type I error). Summing up, results from the linear mixed models analysis suggest that by using mixed linear models it is possible to account for random effects produced by response coordination. In both experiments, including response coordination as a random effect significantly improved model fit, suggesting that accounting for random effects at the pair level allows to reduce substantial biases in analyses (Dittrich et al., 2017). However, since in Experiment 1 response coordination did not mediate the interaction between Correspondence and Condition, the JSE under cooperative instructions cannot be interpreted as the mere consequence of the perturbation produced by a dynamic event in the task environment.

#### RTs Distribution

In order to evaluate the time course of the JSE across experiments, we performed an ANOVA with Condition (Individual vs. Joint), and Correspondence (non-corresponding vs. corresponding) and Bin (1–4) as within-participants factors. In addition, we included Experiment (Exp. 1 vs. Exp. 2) as between-subjects factor. Results are reported in the **Supplementary Material (SM-1)**. In these analyses we observed significant three-way interactions Bin x Condition x Experiment, *F*_1,38_ = 6.07, *p* = 0.001, $\eta_{p}^{2}$ = 0.14, and Experiment x Condition x Correspondence interaction, *F*_1,38_ = 6.66, *p* = 0.014, $\eta_{p}^{2}$ = 0.15. In order to explore in more detail the three-way interactions we performed two separate ANOVAs for Individual and Joint condition, including Correspondence (non-corresponding vs. corresponding) and Bin (1–4) as within-participant factors, and Experiment (Exp. 1 vs. Exp. 2) as between-subjects factor.

Individual condition. The ANOVA revealed a main effect of Bin, *F*_1,38_ = 452.35, *p* < 0.001, $\eta_{p}^{2}$ = 0.92, and a main effect of Correspondence, *F*_1,38_ = 4.86, *p* = 0.034, $\eta_{p}^{2}$ = 0.11. No main effect or significant interaction with Experiment were found, all *F*s < 1.

Joint condition. The ANOVA revealed a main effect of Bin, *F*_1,38_ = 269.29, *p* < 0.001, $\eta_{p}^{2}$ = 0.88, a main effect of Correspondence, *F*_1,38_ = 21.26, *p* < 0.001, $\eta_{p}^{2}$ = 0.36. The main effect of Experiment was significant, *F*_1,38_ = 12.71, *p* = 0.001, $\eta_{p}^{2}$ = 0.25, indicating that participants performed faster under competitive (*M* = 305 ms, *SE* = 7.84 ms) than cooperative instructions (*M* = 344 ms, *SE* = 7.84 ms). No main effect or significant interaction with Experiment were found, all *p*s > 0.08.

#### Trial-by-Trial Modulation and Transition Effects

In order to compare trial-by-trial modulations across experiments, we performed an ANOVA with Condition (Individual vs. Joint), Trial Transition (n-1 Go/ n go vs. n-1 Nogo/ n go), Trial n-1 Correspondence (non-corresponding vs. corresponding), and Trial n Correspondence (non-corresponding vs. corresponding) as within-participant factors. Also for this analysis, Experiment (Exp. 1 vs. Exp. 2) was included as between-subject factor. Results are reported in **Supplementary Material (SM-2)**. In these analyses we observed a marginally significant Condition x Trial n Correspondence x Experiment interaction, *F*_1,38_ = 4.21, *p* = 0.047, $\eta_{p}^{2}$ = 0.10. In order to explore in more detail the three-way interaction, we performed two separate ANOVAs for Individual and Joint condition, including Trial Transition (n-1 Go/ n go vs. n-1 Nogo/ n go), Trial n-1 Correspondence (non-corresponding vs. corresponding), and Trial n Correspondence (non-corresponding vs. corresponding) as within-participant factors, and Experiment (Exp. 1 vs. Exp. 2) as between-subjects factor.

Individual condition. The ANOVA showed a main effect of Trial n-1 Correspondence, *F*_1,38_ = 5.26, *p* = 0.027, $\eta_{p}^{2}$ = 0.12, and a main effect of Trial n Correspondence*, F*_1,38_ = 6.07, *p* = 0.018, $\eta_{p}^{2}$ = 0.14. The interaction between Trial n Correspondence and Trial n-1 Correspondence was also significant, *F*_1,38_ = 45.68, *p* < 0.001, $\eta_{p}^{2}$ = 0.55, as well the three way interaction with Trial Transition, *F*_1,38_ = 49.70, *p* < 0.001, $\eta_{p}^{2}$ = 0.57. Planned comparison showed that trial-by-trial modulations for Nogo/go transitions with a significant 22-ms effect following a corresponding n-1 trial, *t*_39_ = 7.77, p_Bonferroni-corrected_ < 0.001, *d* = 1.23, and a reversed 13-ms effect following a non-corresponding n-1 trial, *t*_39_ = 4.57, p_Bonferroni-corrected_ < 0.001, *d* = 0.72. No trial-by-trial modulations occurred for Go/go transitions, all *p*s > 0.08. No other main effects or interaction were significant, all *p*s > 0.14. No main effect or significant interaction with Experiment were found, all *p*s > 0.17.

Joint Condition. The ANOVA showed a main effect of Trial n Correspondence*, F*_1,38_ = 18.61, *p* < 0.001, $\eta_{p}^{2}$ = 0.33. The interaction between Trial n Correspondence and Trial n-1 Correspondence was also significant, *F*_1,38_ = 115.96, *p* < 0.001, $\eta_{p}^{2}$ = 0.75, as well the three way interaction with Trial Transition, *F*_1,38_ = 14.86, *p* < 0.001, $\eta_{p}^{2}$ = 0.28. Planned comparison showed that trial-by-trial modulations for Nogo/go transitions with a significant 23-ms effect following a corresponding n-1 trial, *t*_39_ = 7.49, p_Bonferroni-corrected_ < 0.001, *d* = 1.18, and a reversed 9-ms effect following a non-corresponding n-1 trial, *t*_39_ = 3.49, p_Bonferroni-corrected_ = 0.001, *d* = 0.55. When trial transition was Go/go, a significant 10-ms effect occurred following a corresponding n-1 trial, *t*_39_ = 3.87, p_Bonferroni-corrected_ = 0.02, *d* = 0.61, and a 2-ms null effect following a non-corresponding n-1 trial, *t*_19_ < 1. The main effect of Experiment was significant, *F*_1,38_ = 12.65, *p* = 0.001, $\eta_{p}^{2}$ = 0.25, indicating that participants performed faster under competitive (*M* = 304 ms, *SE* = 7.80 ms) than cooperative instructions (*M* = 344 ms, *SE* = 7.80 ms). No significant interactions with Experiment were found, all *p*s > 0.10.

#### Response Coordination

**FIGURE 4:**
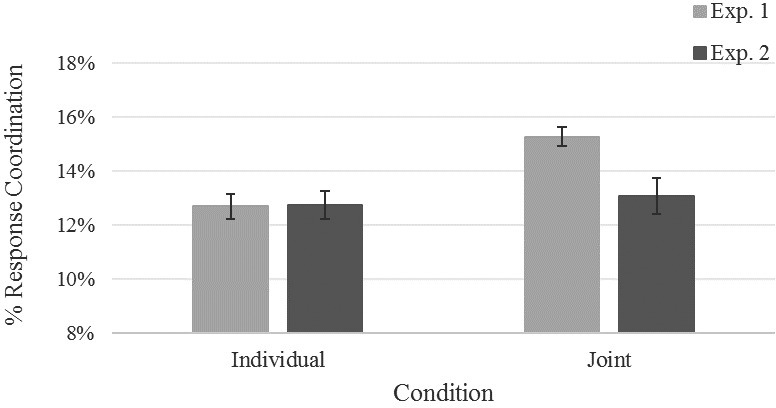
Average percentage of response coordination as a function of Condition (individual vs. joint) in Experiment 1 (light gray bars) and Experiment 2 (dark gray bars). Error bars show standard errors of the means.

In order to assess the effect of goal interdependency (and thus shared representation) in modulating the percentage of response coordination across the two experiments, we conducted an additional analysis to compare data pattern from the two experiments. The proportion of correlated activity was entered into an ANOVA with Condition (Individual vs. Joint) as within-subjects factor and Experiment as between-subjects factor. The analysis revealed a main effect of Condition, *F*_1,18_ = 11.23, *p* = 0.004, $\eta_{p}^{2}$ = 0.38, and a significant two-way interaction with Experiment, *F*_1,18_ = 6.43, *p* = 0.021, $\eta_{p}^{2}$ = 0.26. Two separate one-way ANOVAs indicated that for the Joint condition the percentage of response coordination was higher in Experiment 1 (15.3%) than in Experiment 2 (13.1%), *F* = 8.74, *p* = 0.008, *d* = 1.32. No difference emerged for the Individual condition between the two experiments, *F* < 1 (**Figure 4**).

The comparison of the two experiments showed that when participants performed the task alone, a comparable response coordination between pseudo pairs occurred, independently from the experiment at which they were assigned. In contrast, when they were performing the task side-by-side of another person, response coordination in joint condition increased (relatively to the individual condition) only under cooperation. This latter result indicates that the increased response coordination in the joint Simon Task cannot be explained by the perturbation of temporal and spatial features induced by presence of a second agent performing a task. Our results suggest that positive goal interdependency (shared representation) may be a necessary condition for response coordination and inter-agent response dynamics.

## General Discussion

The present study aimed at examining the role of temporally evolving coupling and inter-agent response dynamics during joint tasks. In two experiments, we investigated the contribution of interpersonal coordination to the emergence of JSE, as an index of shared representations. In both experiments, participants performed a go/no-go Simon task alone and together with another agent in two consecutive sessions. Across experiments, we manipulated goal interdependency by administering cooperative or competitive instructions. In Experiment 1, we instructed participants to cooperate during the social task, while in Experiment 2 participants were required to compete against each other. We examined JSE and response coordination between co-agents as a function of instructed competition or cooperation.

### Shared Representations and Joint Simon Task

Results from the analysis of mean RTs confirmed that when participants performed the go/no-go Simon task alongside another agent, social presence modulated the SE only for the group instructed to cooperate (Experiment 1). On the contrary, no influence of the presence of the co-agent was observed in the group who received competitive instructions (Experiment 2), as indicated by the lack of significant interaction between correspondence and condition (Individual vs. Joint). Our results replicate previous studies showing that when co-agents’ goals are mutually exclusive, the presence of another agent does not affect performance (Iani et al., 2011, 2014). Our results extend previous studies in different ways. First, we directly compared social and individual context within participants. Indeed, previous studies investigating cooperation and competition in the joint Simon task did not include an individual condition as a baseline (Ruys and Aarts, 2010; Iani et al., 2011; Ruissen and de Bruijn, 2016). This allowed us to compare the effect of goal interdependency in the social environment with a baseline performance for each participant in the individual go/no-go task. Sellaro et al. (2013) showed that when the SE emerges in a go/no-go task due to non-social factors (i.e., the extended practice with spatially compatible actions, as typing on a keyboard), then the JSE vanishes in the subsequent task. However, this was not our case: although in the individual condition participants had an empty chair next to them and were aware of the presence of another person in the neighboring room, the magnitude of the SE in the individual condition was comparable across experiments. Results from the distributional analysis do not show any differences in the time course of the SE in the Joint and Individual conditions across the two experiments, as indicated by the lack of four-way interaction. This result is in line with evidence reported by Liepelt et al. (2011) showing that the time course of the JSE is stable across conditions and despite the faster performance under competitive instructions. Moreover, it should be noted that the lack of differences across experiments in the time course suggests that the non-significant JSE under competitive or negative relationships cannot be attributed to faster RTs (Hommel et al., 2009). However, further research is needed to address the relationship between the JSE and response speed. Thus, we can argue that the differential effect of social presence on the JSE between the two social conditions (competitive vs. cooperative) can be explained mainly by the difference in goal interdependency. Second, we investigated the relationship between response coordination and the JSE under cooperative and competitive instructions. Our results showed that response coordination in joint Simon task increased (relative to the individual condition) only under cooperative instructions. Interestingly, when participants were in competition, response coordination of the co-agents was equal to when they performed the task alone. This result suggests that the positive or negative interdependency between co-agents’ goals is reflected not only in the representation of the task (i.e., Iani et al., 2011, 2014) but it also at the sensorimotor timing level. In line with results reported by Liepelt et al. (2011) RTs distribution was comparable between conditions (Individual vs. Joint) and across experiments, suggesting that cooperative and competitive instructions do not affect the temporal dynamics of the JSE. Trial-by-trial effects were comparable across experiments with a significant effect following a corresponding trial, and a non-significant or even reversed after a not corresponding one (Liepelt et al., 2011; Yamaguchi et al., 2018; but see also Ciardo et al., 2018 for results using social cues). Sequential modulations are thought to represent reactive adjustments of control settings (an increase of attention weights on relevant information after experiencing conflict in a non-corresponding trial; e.g., Iani et al., 2009), priming of an earlier stimulus episode (Hommel et al., 2004), or both. Interestingly, we found comparable trial-by-trial modulation in the Individual and Joint condition when the preceding trial was a Nogo trial, i.e., when no response occurred in the Individual condition or the trial required a response of the co-agent in the Joint task. Again, no differences emerged across experiments suggesting that during both cooperative and competitive joint tasks participants represented the co-agents’ S-R associations. Finally, we used punishment avoidance instead of reward to manipulate goal interdependency between co-agents. Our results generalized previous findings showing that the JSE in cooperative vs. competitive condition can be modulated by the need to avoid punishment. Taken together, our results confirm that JSE is elicited by goal sharing between co-agents (Iani et al., 2011) and not by the mere fact that during social task co-agents attend to each other (Ruys and Aarts, 2010).

### Interpersonal Coordination

In line with Malone et al. (2014), work the analysis of temporally emergent response coordination revealed that when participants were instructed to cooperate, the percentage of coordination between co-agents’ responses was higher relative to the individual condition (Experiment 1). Interestingly, this was not true when participants were in competition, as indicated by the lack of difference in the percentage of response coordination across conditions in Experiment 2. The lack of increase of coordination occurred despite the overall speeding up of responses in the joint condition, which is in line with data showing that when performing a Simon task alongside another person, response speed does not correlate with asynchrony between co-agents’ responses (Vesper et al., 2011).

It can be argued that the percentage of response coordination in our study is smaller in size than those reported in previous studies. Indeed, in two different studies, Malone et al. (2013, 2014) reported on average 24 and 33% of response coordination between co-agents for the individual and joint condition, respectively. In our study, we found on average 13% of response coordination for the individual condition and the 15% of correlation between co-agent’s responses in the cooperative condition (Experiment 1, joint condition). The discrepancy between our results and those reported by Malone et al.’s (2013, 2014) could be explained by the fact that in their work, the joint task included 1100 trials. Our joint task comprised only 384 trials. Thus, it is possible that in our study participants did not reach the same amount of response correlation given the lower number of trials, which consisted in a lower number of samples available to create an accurate model of the co-agent’s behavior. However, the choice to include a lower number of trials was motivated by the need to avoid transfer of learning effects typical in spatial compatibility tasks (e.g., Lugli et al., 2013), since we manipulated the social presence (Individual vs. Joint) within participants. In addition, in computing RT time-series, we considered all the data points collected during the task, while Malone and colleagues analyzed only the last 512 responses. By analyzing all the collected responses, we also considered the initial phase of the task during which participants could not have yet coordinated. Future studies should explore in more depth the lack of response coordination in the Joint Simon task under competitive instructions. For instance, by analyzing the structure of RT variability in order to test if during a competitive task responses of co-agents are characterized by nested patterns of variability or by random fluctuations (Malone et al., 2014).

Interestingly, results of Experiment 2 showed that response coordination was not modulated by the competitive social context. By comparing the two experiments, we showed that the lack of difference across conditions in Experiment 2 could not be explained by a difference in the two groups. Indeed, we reported that the percentage of response correlation did not differ across experiments in the Individual condition (12.7% for both experiments). Our results extend Malone et al. (2013, 2014) evidence by showing that when co-agents are in competition, response coordination in the joint Simon Task is comparable to when they perform the go/no-go task alone, despite the dynamic nature of the tasks and their constraints are different. The increase of correlation between co-agents’ responses reported in Experiment 1 cannot be interpreted only as the consequence of the natural tendency to adapt the timing of movements to timing of external events (e.g., Marsh et al., 2009). Indeed, if this was the case, the same pattern should have emerged in Experiment 2. On the contrary, the comparison between the two experiments indicates that in Experiment 2 response coordination was not affected by the mere presence of a competitor acting in the shared setting (i.e., both the screen and the keyboard were shared), suggesting that response coordination emerges when the framework of the task allows co-agents to become an integrated perception-action system.

### Social Context and Coordination – The Case of Cooperation and Competition

Our results suggest that emergence of shared representations is a necessary condition for temporally evolving response coordination but not necessarily a sufficient one. Accordingly, by introducing response coordination as a random effect in mean RTs models, we showed that response coordination did not affect the JSE in the cooperative joint task. Such result is in line with a recent study by Dittrich et al. (2017), showing that including random effect at the pair level increases model fitting and reduces potential biases driven by differences across pairs. The current findings give hints about the relation between shared representations or self-other integration indexed by JSE, sensorimotor coordination, and goal sharing and how these mechanisms are orchestrated to reach efficient joint action. Specifically, our results suggest that response coordination between co-agents does not account for JSE. However, they also highlight the importance of taking into account response coupling between co-agents when investigating the nature of the JSE (Dittrich et al., 2017). The percentage of coupling and the JSE might be considered as two independent elements supporting effective joint action. Keller et al. (2016) proposed that joint action outcome results from the integration and segregation of internal models of the self and of others. The authors proposed that during joint action, although goals are represented as shared, in order to guide the joint performance, a distinction between self and other internal model is preserved to allow each co-agent to keep control over their action planning and execution. This facilitates co-agents to anticipate, attend and adapt to each other in real time, resulting into a precise and flexible interpersonal coordination (c.f. Keller et al., 2016). It is plausible that in our study, when co-agents’ goals were positively related, shared goal representation may have promoted the integration of self and other models. As a result, in the joint action model both alternative of response were represented. Self-other integration allowed co-agents to attend and adapt their performance to each other’s sensorimotor timing, resulting in response coordination. In contrast, when the co-agents were in competition, the lack of shared representation may have favored self-other segregation. Thus, the resulting joint model did not include co-agent’s alternative of response. Therefore co-agents did not consider other’s behavior, but focused on stabilizing their own performance (i.e., increasing the response speed) in order to suppress the sensorimotor interference generated by co-agent’s action timing. Then, as a final result no response coordination emerged.

Our results are limited to joint tasks based on discrete non-rhythmic actions. However, they highlight the importance of competitive interactions in the context of understanding how different mechanisms support joint action. If we suppose that goal interdependency between co-agents may vary on a dipole between positive and negative relation, then we can assume that when cooperation is not explicitly requested, the shared nature of the task (i.e., context, setting, and space) prompts the integration of self and other models into a joint model. As a result, when others do not explicitly interfere with our goals, by default we perceive positive interdependence with them (Ruys and Aarts, 2010; Iani et al., 2011), and coordinate with them at the sensorimotor level (Malone et al., 2013, 2014) even if their actions have no direct consequences on our next action, as in the case of the joint Go/nogo Simon task. Future studies should address the relation between coordination and the emergence of the JSE by de-personalizing the goal of the task. For instance, synchronization with external and variable events generated by human or artificial agents can be examined (e.g., a Humanoid robot, see Wykowska et al., 2016; Wiese et al., 2017), as perceived natural/intentional vs. artificial agency may moderate self-other integration in the Joint Simon task. In addition, future studies need to address the role of visual access to co-agent’s action for the emergence of response coordination during joint tasks.

To conclude, the present study was designed to investigate the contribution of interpersonal entrainment to the emergence of shared representations by comparing response coordination in a joint Simon task during cooperation and competition. Our results show that emerging coordination increases during joint action only if co-agents’ goals are shared, but not when co-agents’ goals are mutually exclusive. The results show that interpersonal coordination requires the emergence of shared representations or self-other integration, indexed by JSE. Therefore, in joint action shared representations seem to be a necessary condition for interpersonal coordination, but not sufficient one.

## Author Contributions

FC conceived, designed and performed the study, analyzed the data, discussed and interpreted the results, and wrote the manuscript. AW conceived and designed the study, discussed and interpreted the results, and wrote the manuscript. All authors reviewed the manuscript.

## Conflict of Interest Statement

The authors declare that the research was conducted in the absence of any commercial or financial relationships that could be construed as a potential conflict of interest.

## Footnotes

1 According to the classification of spatial compatibility effects proposed by Donders (1969), the SE typically reported in the Joint Go/Nogo Simon task belongs to group “c,” including spatial-compatibility effects emerging in tasks in which the spatial nature of the stimulus is task-irrelevant.

2 Note that when decomposing reaction times variability, it is possible to identify three types of temporal structures. White noise indicates that the temporal variability in an action sequence is generated by unsystematic or unrelated changes from trial to trial. Brown noise corresponds to a stochastic function, indicating that each subsequent action is a function of the previous action to which a random increment is added. Pink noise is a mixture of randomness and rigidity, and it is typical of interaction dominant (complex) systems across multiple time scales. For example, it has been suggested that pink noise reflects emergent coordination between cognitive processes and behavior (e.g., Van Orden et al., 2003).

## Supplementary Material

The data related to this study can be accessed online at https://doi.org/10.5281/zenodo.1421280.
