# Supplementary material for: Response Coordination Emerges in Cooperative but Not Competitive Joint Task
Source: Front Psychol. 2018 Oct 9;9:1919. doi: 10.3389/fpsyg.2018.01919 (PMC6189413; doi:10.3389/fpsyg.2018.01919)
Supplement: Supplementary file 1 [file Data_Sheet_1.PDF]

Supplemental Material.

Response coordination emerges in cooperative but not competitive joint task

Francesca Ciardo[1*] and Agnieszka Wykowska[1, 2]

[1] Istituto Italiano di Tecnologia, Genoa, Italy

[2] Luleå University of Technology, Luleå, Sweden

Correspondence to:

Francesca Ciardo
Istituto Italiano di Tecnologia
Centre for Human Technologies
Via E. Melen, 83
16040 Genova, Italy
E-mail: francesca.ciardo@iit.it

SM-1. Comparison across Experiments: RTs Distributions.

In order to evaluate the time course of the JSE across experiments, we performed an ANOVA with Condition (Individual vs. Joint), and Correspondence (non-corresponding vs. corresponding) and Bin (1 to 4) as within-participant factors. In addition, we included Experiment (Exp.1 vs. Exp. 2) as between-subjects factor. Mauchly's test indicated that the assumption of sphericity had been violated for the main factor Bin ($\chi 2$ = 213.31, $p < .001$), therefore degrees of freedom were corrected using Greenhouse-Geisser estimates of sphericity ($\varepsilon = 0.36$). Results are reported in Table 1. When necessary, within-subjects comparisons were performed using paired samples t-tests. Significance thresholds were corrected for the number of comparisons (Bonferroni correction). The ANOVA revealed a main effect of Bin, $F_{1.09, 41.43} = 486.50$, $p < .001$, $\eta_p^2 = .93$, indicating that RTs increase across quartiles. The main effect of Condition was significant, $F_{1, 38} = 14.04$, $p < .001$, $\eta_p^2 = .27$, indicating faster RTs in the Joint (M = 325 ms, SE = 5.55 ms) compare to the Individual condition (M = 343 ms, SE = 6.84 ms). It was qualified by a two-way interaction with Experiment, $F_{1, 38}$ = 11.44, p = .002, $\eta_p^2 = .23$. One-way ANOVAs showed that for the Joint condition RTs were faster in Exp.2 (M = 305 ms, SE = 7.84 ms) than in Exp.1 (M = 344 ms, SE = 7.984 ms), F_{39} =12.71, p= .001, $\eta_p^2 = 0.25$. No differences across experiments emerged for the Individual condition, F<1. The main effect of Correspondence was significant, $F_{1, 38} = 15.66$, $p < .001$, $\eta_p^2 = .29$, indicating a 5 ms SE (corresponding trials: M = 331 ms, SE = 5.75 ms; non-corresponding trials: M= 336 ms, SE = 5.81 ms). The interaction between Correspondence and Condition was also significant, $F_{1, 38} = 5.09$, p = .030, $\eta_p^2 = .12$. Planned comparison showed that non-corresponding trials were faster (M = 321 ms, SE = 6.28 ms) than non-corresponding trial (M = 328 ms, SE = 6.47 ms) both in the Joint, $t_{39} = 4.49$ $p_{Bonferroni\text{-}corrected} < .001$, d = .71, and in the Individual condition (corresponding trials: M = 341 ms, SE = 6.75 ms; non-corresponding trials: M = 345 ms, SE = 6.88 ms), t_{39} = 2.21, $p_{Bonferroni\text{-}corrected} = .03$, d = .35. We compare across conditions the SE computed as the difference between non-corresponding and corresponding trial. Paired t-test showed that the 7 ms SE in the Joint condition differed from the 4 ms SE reported in the Individual condition, $t_{39} = 2.11$, $p_{Bonferroni\text{-}corrected} = .04$, d = .33. The main effect of Experiment was marginally significant, $F_{1, 38} = 4.09$, p = .050, $\eta_p^2 = .10$, indicating that overall participants performed faster (M= 322, SE= 8.11) in Exp.2 than in Exp.1 (M= 345, SE= 8.11). The three-way interaction Bin x Condition x Experiment was significant, $F_{1, 38} = 6.07$, p= .001, $\eta_p^2 = .14$, as well the Experiment x Condition x Correspondence interaction, $F_{1, 38} = 6.66$, p = .014, $\eta_p^2 = .15$. In order to explore more in details the three way interactions involving both the factor Experiment we performed two separate ANOVAs for Individual and Joint condition, including Correspondence (non-corresponding vs. corresponding) and Bin (1 to 4) as within-participant factors, and Experiment (Exp.1 vs. Exp. 2) as between-subjects factor

SM-2. Comparison across Experiments: Trial-by-trial modulation and Transition effects.

In order to compare trial-by-trial modulations across experiments, we performed an ANOVA with Condition (Individual vs. Joint), Trial Transition (n-1 Go/ n go vs. n-1 Nogo/ n go), Trial n-1 Correspondence (non-corresponding vs. corresponding), and Trial n Correspondence (non-corresponding vs. corresponding) as within-participant factors. Also for this analysis, Experiment (Exp.1 vs. Exp.2) was included as between-subject factor. Results are reported in Supplementary Material (SM-Table 2). When necessary, within-subjects comparisons were performed using paired samples t-tests. Significance thresholds were corrected for the number of comparisons (Bonferroni correction). The ANOVA showed a main effect of Condition, $F_{1, 38}$ = 14.33, p = .001, $\eta_p^2 = .27$, indicating faster RTs in the Joint (M = 324 ms, SE = 5.51 ms) compare to the Individual condition (M = 342 ms, SE = 6.82 ms). This main effect entered into a significant two-way interaction with Experiment, $F_{1, 38} = 11.00$, p =.002, $\eta_p^2 = .23$. One-way ANOVAs showed that for the Joint condition RTs were faster in Exp.2 (M = 304 ms, SE = 7.80 ms) than in Exp.1 (M = 344 ms, SE = 7.80 ms), F_{39} =12.65, < .001, $\eta_p^2 = 0.25$. No differences across condition emerged in Exp.1, F< 1. The interaction Condition x Trial n-1 Correspondence was significant, $F_{1, 38} = 8.23$, p =.007, $\eta_p^2 = .18$. Planned comparison showed that Trial N-1 correspondence affected RTs in the Individual condition only, with RTs faster following non-corresponding trial n-1(M = 341 ms, SE = 6.68 ms) than following a corresponding trial n-1 (M = 344 ms, SE = 6.89 ms), $t_{39} = 2.32$, $p_{Bonferroni\text{-}corrected} = .03$, d =1.38. No differences were in RTs emerged according to trial n-1 correspondence in the Joint condition (non-corresponding n-1 trials: M = 325 ms, SE = 6.23 ms; corresponding n-1 trials: M = 323 ms, SE = 6.37 ms), $t_{39} = 1.5$, $p_{Bonferroni\text{-}corrected} = .14$, d = .24. The interaction between Trial n Correspondence and Trial n-1 Correspondence was also significant, $F_{1, 38} = 145.89$, $p < .001$, $\eta_p^2 = .79$, as well the three way interaction Trial Transition x Trial n-1 Correspondence x Trial n Correspondence, $F_{1, 38} = 51.75$, $p < .001$, $\eta_p^2 = .58$. Planned comparison showed that trial-by-trial modulations for Nogo/go transitions with a significant 23-ms effect following a corresponding n-1 trial, $t_{39} = 9.32$, $p_{Bonferroni\text{-}}$

$_{corrected}$ < .001, d =1.44, and a reversed 11-ms effect following a non-corresponding n-1 trial, t_{39} =5.41, $p_{Bonferroni-corrected}$ < .001, d = 0.86. When trial transition was Go/go a significant 6-ms effect occurred following a corresponding n-1 trial, t_{39} = 3.27, $p_{Bonferroni-corrected}$ = .002, d = .52, and a 3-ms not-significant effect following a non-corresponding n-1 trial, t_{39}= 1.83, p= .075, d= .29. The main effect of Trial n Correspondence was significant, $F_{1, 38}$ = 16.44, p < .001, η_p^2= .30, indicating a 5ms SE (corresponding trials: M = 331 ms, SE = 5.70 ms; non-corresponding trials: M = 336 ms, SE = 5.78 ms). The main effect of Experiment was marginally significant, $F_{1, 38}$ = 4.08, p = .050, η_p^2= .10, as well the three-way interaction between Condition xTrial n Correspondence x Experiment was significant, $F_{1, 38}$ = 4.21, p = .047, η_p^2= .10. In order to explore more in details the three way interaction involving the factor of Experiment we performed two separate ANOVAs for Individual and Joint condition, including Trial Transition (n-1 Go/ n go vs. n-1 Nogo/ n go), Trial n-1 Correspondence (non-corresponding vs. corresponding), and Trial n Correspondence (non-corresponding vs. corresponding) as within-participant factors, and Experiment (Exp.1 vs. Exp. 2) as between-subjects factor."

Table SM.1. Results of the Analysis of Variance (ANOVA) on mean RTs with Condition (Individual vs. Joint), and Correspondence (non-corresponding vs. corresponding) and Bin (1 to 4) as within-participants factors; and Experiment (Exp.1 vs. Exp.2) as between-participants factor.

**Main effects and interactions**	**F**	**p**	η_p^2
Experiment	4.090	0.050	0.097
Bin*	486.500	<0.001	0.928
Condition	14.044	0.001	0.227
Correspondence	15.660	<0.001	0.292
Condition x Experiment	11.437	0.002	0.231
Condition x Bin*	2.528	0.117	0.062
Condition x Correspondence	5.093	0.030	0.102
Bin x Experiment	3.116	0.029	0.076
Bin x Correspondence*	0.216	0.726	0.006
Correspondence x Experiment	0.272	0.605	0.007
Condition x Bin x Experiment	6.072	0.001	0.138
Bin x Correspondence x Experiment	0.191	0.902	0.005
Condition x Bin x Correspondence*	0.057	0.902	0.001
Condition x Correspondence x Experiment	6.660	0.014	0.149
Condition x Bin x Correspondence x Experiment	0.912	0.438	0.023
* Mauchly's test of sphericity indicates that the assumption of sphericity is violated (p < .05). Reported values are estimated using the GreenhouseGeisser correction.			

Table SM.2. Results of the Analysis of Variance (ANOVA) on mean RTs with Condition (Individual vs. Joint), Trial Transition (n-1 Go/ n go vs. n-1 Nogo/ n go), Trial n-1 Correspondence (non-corresponding vs. corresponding), and Trial n Correspondence (non-corresponding vs. corresponding) as within-participant factors, and Experiment (Exp.1 vs. Exp.2) as between-participants factor.

**Main effects and interactions**	**F**	**p**	**η_p^2**
Experiment	4.082	0.050	0.097
Condition	14.332	0.001	0.274
Trial Transition	0.049	0.826	0.001
Trial n-1 Correspondence	0.856	0.361	0.022
Trial n Correspondence	16.438	< 0.001	0.302
Condition x Experiment	11.002	0.002	0.225
Condition x Trial Transition	0.167	0.685	0.004
Condition x Trial n-1 Correspondence	8.226	0.007	0.178
Condition x Trial n Correspondence	2.810	0.102	0.069
Trial Transition x Experiment	0.652	0.424	0.017
Trial Transition x Trial n-1 Correspondence	1.003	0.323	0.026
Trial Transition x Trial n Correspondence	0.591	0.447	0.015
Trial n-1 Correspondence x Experiment	0.151	0.700	0.004
Trial n-1 Correspondence x Trial n Correspondence	145.886	<0 .001	0.793
Trial n Correspondence x Experiment	0.421	0.520	0.011
Condition x Trial Transition x Experiment	1.146	0.291	0.029
Condition x Trial Transition x Trial n-1 Correspondence	0.390	0.536	0.010
Condition x Trial Transition x Trial n Correspondence	0.001	0.974	0.000
Condition x Trial n-1 Correspondence x Experiment	0.512	0.479	0.013
Condition x Trial n Correspondence x Experiment	4.206	0.047	0.100
Condition x Trial n-1 Correspondence x Trial n Correspondence	1.620	0.211	0.041
Trial Transition x Trial n-1 Correspondence x Experiment	0.017	0.898	0.000
Trial Transition x Trial n-1 Correspondence x Trial n Correspondence	51.754	<0.001	0.577
Trial Transition x Trial n Correspondence x Experiment	0.098	0.756	0.003
Trial n-1 Correspondence x Trial n Correspondence x Experiment	0.322	0.574	0.008
Condition x Trial Transition x Trial n-1 Correspondence x Experiment	0.026	0.872	0.001
Condition x Trial Transition x Trial n Correspondence x Experiment	0.663	0.420	0.017
Condition x Trial n-1 Correspondence x Trial n Correspondence x Experiment	0.031	0.861	0.001
Condition x Trial Transition x Trial n-1 Correspondence x Trial n Correspondence	2.732	0.107	0.067
Trial Transition x Trial n-1 Correspondence x Trial n Correspondence x Experiment	0.208	0.651	0.005
Condition x Trial Transition x Trial n-1 Correspondence x Trial n Correspondence x Experiment	0.276	0.603	0.007
